# Outbreak Investigation: Jamestown Canyon Virus Surveillance in Field-Collected Mosquitoes (Diptera: Culicidae) From Wisconsin, USA, 2018–2019

**DOI:** 10.3389/fpubh.2022.818204

**Published:** 2022-04-21

**Authors:** Melissa R. Farquhar, Nicholas B. Thrun, Bradley J. Tucker, Lyric C. Bartholomay

**Affiliations:** ^1^Midwest Center of Excellence for Vector-Borne Disease, University of Wisconsin-Madison, Madison, WI, United States; ^2^Department of Pathobiological Science, School of Veterinary Medicine, University of Wisconsin-Madison, Madison, WI, United States; ^3^Department of Entomology, College of Agriculture and Life Sciences, University of Wisconsin-Madison, Madison, WI, United States

**Keywords:** *Aedes provocans*, arbovirus, vertical transmission, spring *Aedes*, Midwest USA

## Abstract

During the summers of 2017–2019, 60 human cases of Jamestown Canyon virus-associated disease were reported in the State of Wisconsin, U.S.A; by comparison, there were 28 cases in the 5 years prior. Jamestown Canyon virus (JCV, Peribunyaviridae: *Orthobunyavirus*) is a zoonotic, mosquito-borne virus that is endemic throughout North America. The proposed transmission cycle for JCV involves horizontal transmission by a variety of mammal-feeding mosquito species and deer hosts, and transseasonal maintenance by vertical transmission in *Aedes* mosquito species. Although some of the earliest work on JCV transmission and disease was done in Wisconsin (WI), little is known about the spectrum of mosquitoes that are currently involved in transmission and maintenance of JCV, which is key to inform the approach to control and prevent JCV transmission, and to understand why case numbers have increased dramatically in recent years. Therefore, we undertook an intensive surveillance effort in Sawyer and Washburn counties, WI between April and August of 2018 and 2019, in an area with a concentration of JCV human cases. Larval and adult stages of mosquitoes were surveyed using larval dippers and emergence traps, light traps, resting boxes, a Shannon-style trap, and backpack aspirator. In total, 14,949 mosquitoes were collected in 2018, and 28,056 in 2019; these specimens represent 26 species in 7 genera. Suspect vector species were tested for JCV by polymerase chain reaction (PCR); of 23 species that were tested, only *Aedes provocans* yielded JCV positive results. In 2018, a single pool of *Ae. provocans* tested positive. In 2019, with more focused early season surveillance, we detected JCV in 4 pools of adult mosquitoes, and one pool that consisted of lab-raised adults that were collected as larvae. Material from all of these PCR-positive samples also yielded infectious virus in cell culture. Overall, these data provide new insight into the seasonality and habitat preferences for 26 mosquito species in Northern WI, which will be useful to inform future surveillance efforts for JCV. The results underscore the importance of *Ae. provocans* as a vector species involved in transseasonal maintenance of JCV in this region.

## Introduction

Jamestown Canyon Virus (JCV) (Peribunyaviridae: *Orthobunyavirus*) is a California serogroup (CEV) virus first isolated from field-collected mosquitoes (Diptera: Culicidae) in Colorado in 1961 (1). The earliest evidence for JCV-associated disease in humans was documented in 1963 through a serosurvey of forest workers in north-central Wisconsin (WI) (Bayfield, Vilas, and Jackson Counties), in which four individuals experienced febrile illnesses during the summer and had a demonstrated increase in neutralizing antibody to CEV (2). Neuroinvasive disease associated with JCV was first observed in September 1980, when an 8-year-old girl from Southwestern Michigan developed a non-specific febrile illness followed by a coma (3). According to the Centers for Disease Control and Prevention, 15 cases of JCV-associated disease occur on average annually. From 2010 to 2019, 225 cases of JCV were recorded across the United States, and almost half (111 cases) of these occurred in Wisconsin (4). Matkovic et al. suggest that JCV is widespread in Wisconsin residents (5). However, there was a marked increase in JCV cases from 2017 to 2019, during which epidemic levels (43, 23, and 15 cases, respectively) of JCV were confirmed by the Wisconsin Department of Health Services (6).

The transmission cycle for Jamestown Canyon virus involves transseasonal maintenance by vertical and transstadial transmission, and seasonal maintenance by horizontal transmission between mammalophilic mosquitoes and deer hosts, with spill-over into humans (7). Indeed early evidence of this linkage came from WI, because neutralizing antibody to JCV was detected in Wisconsin white-tailed deer in November 1969, and infectious viremia was confirmed from a sentinel white-tailed deer in May 1971 (8, 9). Subsequent serosurveys of deer in the Midwestern U.S. and Canada revealed high seropositivity to JCV, indicating that these animals have a long-term, protective antibody response to subsequent exposure (10–12). However, there is reason to doubt they are the sole mammalian species contributing to horizontal transmission because there is likely a very short window during which a deer could be viremic. Serosurveys of deer in Michigan indicated that fawns likely have protective maternal antibodies for the first season of mosquito exposure, which wanes such that yearlings are susceptible to the virus the following spring, but rapidly seroconvert upon exposure to JCV-infected mosquitoes (10).

Jamestown Canyon virus transmission does not involve a 1:1 virus:primary vector interaction; in fact, at least 26 species of mosquito across multiple genera in North America have been implicated in JCV transmission based on virus isolation from field-collected mosquitoes (13). A wide variety of *Aedes* spp., as well as *Anopheles punctipennis* and *Coquillettidia perturbans*, are susceptible to infection in laboratory settings. There is evidence that *Aedes stimulans, Ae*. *provocans, Ae*. *canadensis, Ae*. *epactius, Anopheles punctipennis*, and *Coquillettidia perturbans* are susceptible to infection and exhibit horizontal transmission of the virus, with an extrinsic incubation period ranging from 14 to 21 days (14–16). Furthermore, there is evidence for vertical transmission of JCV in *Ae*. *triseriatus, Ae*. *stimulans*, and *Ae*. *provocans*, based on isolation of JCV from field-caught adult males, or from eggs and larvae reared to adults in the lab (15, 17, 18). Because JCV cases occur from spring—fall months, it is likely that early spring *Aedes* species are involved in transseasonal maintenance, and longer-lived or mosquito with multiple generations are involved in horizontal transmission and spill-over to human hosts.

Based on our knowledge about mosquito fauna present in Wisconsin, and the species of mosquito that consistently meet all of the criteria for vector incrimination, *Ae*. *canadensis, Ae*. *provocans, Ae*. *stimulans, An*. *punctipennis*, and *Cq*. *perturbans* were of particular interest in our surveillance efforts. Indeed during the summer of 1965, JCV was isolated from Wisconsin *Aedes stimulans* group and *Aedes communis* group mosquitoes (19). Because much of the work to understand JCV transmission dynamics in WI was done in the 1960s, and there was a distinct surge in human cases of JCV-associated disease beginning in 2017, we undertook a concentrated surveillance effort around a cluster of case sites in the Hayward, WI area to understand what species of mosquito are involved in transmission and transseasonal maintenance of JCV.

## Materials and Methods

### Study Site Selection

Mosquitoes were collected on the Lac Courte Oreilles (LCO) Reservation, Lac Courte Oreilles Band of Lake Superior Chippewa, Ojibwe Nation, in Sawyer County, WI, and at a nearby case site in Washburn County, WI (see Supplementary Figure 1). For reference regarding surveillance logistics, these surveillance sites are an approximately 4.5 h drive from the authors' laboratory at the University of Wisconsin-Madison. Surveillance took place within 20 km of five human JCV cases confirmed in the years 2016–2018, including 2 sites where we were able to do rapid case site investigations based on presumptive (later confirmed) case data. Surveyed sites in 2018 were in a 20 km radius of Grindstone Lake, Sawyer County, coordinates 45.93606, −91.41505. During 2019, we adjusted our collection efforts to focus on monitoring early season vectors in areas near where JCV was detected in 2018. Permissions to trap were secured with colleagues at Indian Health Services, the Lac Courte Oreilles (LCO) Community Health Center, the LCO Ojibwe College, and Sawyer County Public Health. Surveyed sites in 2019 were in a 10 km radius of Grindstone Lake, Sawyer County, coordinates 45.93606, −91.41505. Because we were working with state, local, and jurisdictional health agencies, and because much of our sampling was done on private residences, more precise geolocation of sampling sites is sensitive information that we do not provide herein. Representative images of adult and immature collections sites are provided in Supplementary Figure 1.

### Mosquito Collections, 2018

Mosquitoes were collected once weekly during epidemiological weeks 23, 25, 26, 28, 30, 33, 34, 35, and 38 from June to September. Adult mosquitoes were collected using a carbon dioxide baited Centers for Disease Control (CDC) miniature light trap (John W. Hock Company), alfalfa infusion baited CDC gravid trap (John W. Hock Company), carbon dioxide baited BG-Sentinel 2 (Biogents), and a modified CDC backpack aspirator (John W. Hock Company). Centers for Disease control miniature light traps were suspended ~1.5 m off the ground from vegetation in forested areas. Gravid and BG traps were placed at ground level in shaded locations near vegetation. All adult traps were operated overnight and mosquito samples collected the following morning. Adult resting mosquitoes were collected from vegetation at suspected human case sites with a modified CDC backpack aspirator. Upon collection, trap contents were frozen on dry ice and transported to the laboratory for storage at −80°C. Immature mosquitoes were collected from tires and puddles using a Bioquip mosquito dipper (Bioquip) (see Supplementary Figure 2).

### Mosquito Collections, 2019

Mosquitoes were collected weekly during epidemiological weeks 16, 17, 18, 20 (2 nights), 21 (3 nights), 23, 24, 25, 26, 28, 31, 34, and 36 between April and September. In an effort to increase the number of early season vector species tested, multiple nights of collections were done with host-seeking traps during weeks 20 and 21. Larvae were collected at multiple locations: three habitats of ephemeral ponds alongside county road NN, a permanent water body near the adult collection site, and from a private property near Spring Lake, WI that had both permanent water bodies, such as ponds dominated by bladderworts, and ephemeral pools (see Supplementary Figure 1).

Host-seeking adult mosquitoes were collected using carbon dioxide-baited CDC miniature light trap (John W. Hock Company) and BG-Sentinel 2 (Biogents) traps baited with carbon dioxide operated as outlined for 2018. Upon collection, trap contents were frozen on dry ice and transported to the laboratory for storage at −80°C. Other methods for adult mosquito collection included the use of resting boxes, a Shannon trap baited with UV-light and a modified CDC backpack aspirator (John W. Hock Company) (20). Floating emergence traps were set in larval habitats during weeks 17–21, with the exception of week 19. These traps were monitored daily and newly eclosed mosquitoes collected, frozen on dry ice and transported to the laboratory for storage at −80°C. Immature mosquitoes were collected with Bioquip mosquito dipper (Bioquip) and with the Aquatic Light Trap (Bioquip) during weeks 16–21 (see Supplementary Figure 2). Larvae and pupae were reared in 1.5 L of deionized water in enamel pans at 10°C with 12:12 (L:D) through May 15, and then switched to 20°C with 16:8 (L:D). Laboratory reared adults were transferred to −80°C for storage prior to identification.

### Mosquito Identification

All adult mosquitoes were sorted on a cold table (laboratory chill table, Bioquip) and specimens identified using Darsie and Ward and an unpublished key for dark-legged Wisconsin *Aedes* spp. developed by these authors. Mosquitoes were separated and pooled with a maximum of 50 individuals in 2 ml tubes by sex, species, date, and location of collection. Only female adult mosquitoes were identified and tested for JCV to understand horizontal transmission potential. To screen for vertical transmission of virus, both male and female mosquitoes were identified and tested from immature collections. All specimen identifications were made to species level except for the following taxa for which adult females are insufficiently characterized in the literature: *Ae. stimulans* group mosquitoes were pooled together, *Ae. abserratus* and *Ae. punctor* were pooled together as *Ae. punctor* group, and *Cx. restuans* and *Cx. pipiens* were combined.

### Virus Diagnostics

Mosquito pools were processed to facilitate PCR diagnostics and recovery of “live virus” for pools that yielded a PCR positive result. Each pool was suspended in 1 ml of Dulbecco's Modified Eagle's Medium (DMEM) with 10% heat-inactivated Fetal Bovine Serum, 1% L-glutamine, and 1% Penicillin/Streptomycin. Mosquitoes were homogenized and then centrifuged at 4°C for 5 min at 12,000 rcf. One hundred microliters of supernatant was then added to a 1.5 ml tube containing 300 μl of viral RNA buffer from the Quick-RNA Viral Kit (Zymo Research) and the contents were subjected to RNA extraction according to the manufacturer's instructions. Reverse transcription PCR was used for JCV RNA detection using the following primers: JCS63C (5′-CCTGGTTGATATGGGAGATTTGGTTTTC-3′) and JCS667V (5′-TCTTCTGCGCCATCCACTTCTCTG-3′) (21). Amplification cycles were as follows: 1 × 50°C for 30 min, 1 × 95°C for 2 min, 35 × (95°C for 10 s, 60°C for 10 s, 72°C for 1 min), 1 × 72°C for 2 min. SuperScript III^TM^ One-Step RT-PCR with Platinum® *Taq* was used (Invitrogen) with the following concentration per 1x reaction: 5.3 μl DNase free water, 7.5 μl 2x reaction buffer, 0.3 μl of each primer, 0.6 μl of *Taq*, and 1 μl of sample to test. Gels made with Sodium Borate buffer solution mixed with agarose were used for band visualization under UV-light. Complementary DNA from JCV isolate 3352-17 from Connecticut was used as the JCV positive control (kindly provided by Dr. Philip Armstrong, Connecticut Agricultural Experiment Station). Sanger sequencing performed by the UW Biotechnology Center was used to confirm the sequence of the amplicon in JCV positive pools. Jamestown Canyon virus positive mosquito pools were then propagated in Vero cell culture following established protocols to test for cytopathic effects as evidence of live virus (22). Because of available resources for PCR testing, some mosquito pools (*n* = 273) that were collected and identified in 2018 were not tested in-house, but instead were sent to the Centers for Disease Control and Prevention in Ft. Collins, Colorado for virus testing via cell culture using established protocols (23).

## Results

In response to outbreak-level numbers of JCV human cases, we conducted targeted surveillance for JCV vectors during 2018–2019 in Sawyer and Washburn counties, Wisconsin, U.S.A. Human JCV case data obtained through correspondence with local and state health officials were used to inform collection sites and trapping efforts in 2018. The objective for 2018 surveillance was to determine the number and quantity of suspected vector species present near human case sites and whether any of these species were positive for JCV. A total of 13,751 adult female mosquitoes were combined into 438 pools. These were collected over 73 trap nights (63 CDC light trap nights, 5 BG sentinel trap nights, and 5 gravid trap nights). Collections were made from June to September (epidemiological weeks 23, 25, 26, 28, 30, 33, 34, 35, and 38).

The results from 2018 informed collection site choice for 2019. Efforts were shifted to span a longer duration of the season and thereby increase the quantity of both adult and immature woodland “spring *Aedes* mosquitoes,” that emerge early in the spring months (24). A total of 23,053 adult female mosquitoes combined into 565 pools were collected in 2019 over 197 trap nights (173 CDC, 24 BGS). Collections were made between April-September (epidemiological weeks 16, 17, 18, 20, 21, 23, 24, 25, 26, 28, 31, 34, and 36). A total of 5,003 mosquitoes were collected as larvae, pupae, or newly emerged and nulliparous between April-June (epidemiological weeks 16–23, see Table 1). The types of habitats where larvae were found, including ephemeral pools, permanent water bodies, small ponds and tires, are described in Table 2, and pictured in Supplementary Figure 1. These were combined by species in 308 pools that were tested for JCV infection.

**Table 1:** Total number of immature and newly emerged mosquitoes collected during epidemiological weeks 16–23 2019 in Sawyer and Washburn Counties, WI, U.S.A.

**Species**	**Week of the year**						**Total**
	**16**	**17**	**18**	**20**	**21**	**23**	
*Ae. canadensis*	3	116	206	55	132	0	512
*Ae. cinereus*	0	165	1,325	175	226	0	1,891
*Ae. communis* cpx	3	6	13	13	6	0	41
*Ae. diantaeus*	0	1	15	1	8	0	25
*Ae. implicatus*	0	0	0	3	0	0	3
*Ae. intrudens*	5	31	70	75	32	0	213
*Ae. j. japonicus*	0	0	0	0	0	16	16
*Ae. provocans*	4	22	362	518	**680**	0	1,586
*Ae. punctor* group	0	42	18	11	35	0	106
*Ae. sticticus*	0	8	38	19	3	0	68
*Ae. stimulans* group	0	52	170	43	247	0	512
*Ae. triseriatus*	0	0	0	0	1	25	26
Ae. vexans*	0	3	0	0	1	0	4
Total	15	446	2,217	913	1,371	41	5,003

One pool (n = 42) of Ae. provocans collected during week 21 tested positive for JCV. Species that were collected but not tested for JCV are denoted with ^*^.

*The value in bold indicates that a sample from this week tested positive for JCV*.

**Table 2:** Total number of immature mosquitoes collected in Sawyer County in 2019, according to larval habitat.

**Species**	**Ephemeral pools**	**Permanent water body (marsh, pond)**	**Small ponds and/or ephemeral pools**	**Tires**	**Total**
*Ae. canadensis*	454	42	16	0	512
*Ae. cinereus*	185	1,697	9	0	1,891
*Ae. communis* cpx	41	0	0	0	41
*Ae. diantaeus*	21	4	0	0	25
*Ae. implicatus*	3	0	0	0	3
*Ae. intrudens*	207	2	4	0	213
*Ae. japonicus*	0	0	0	16	16
*Ae. provocans*	1,523	3	60	0	1,586
*Ae. punctor* group	65	8	33	0	106
*Ae. sticticus*	68	0	0	0	68
*Ae. stimulans* group	182	235	95	0	512
*Ae. triseriatus*	0	0	0	25	25
Ae. vexans*	4	0	0	0	4
Total	2,752	1,991	218	41	5,002

*Representative images of habitat types are shown in Supplementary Figure 1*.

Species that were collected but not tested for JCV are denoted with ^*^.

**Figure 1:**
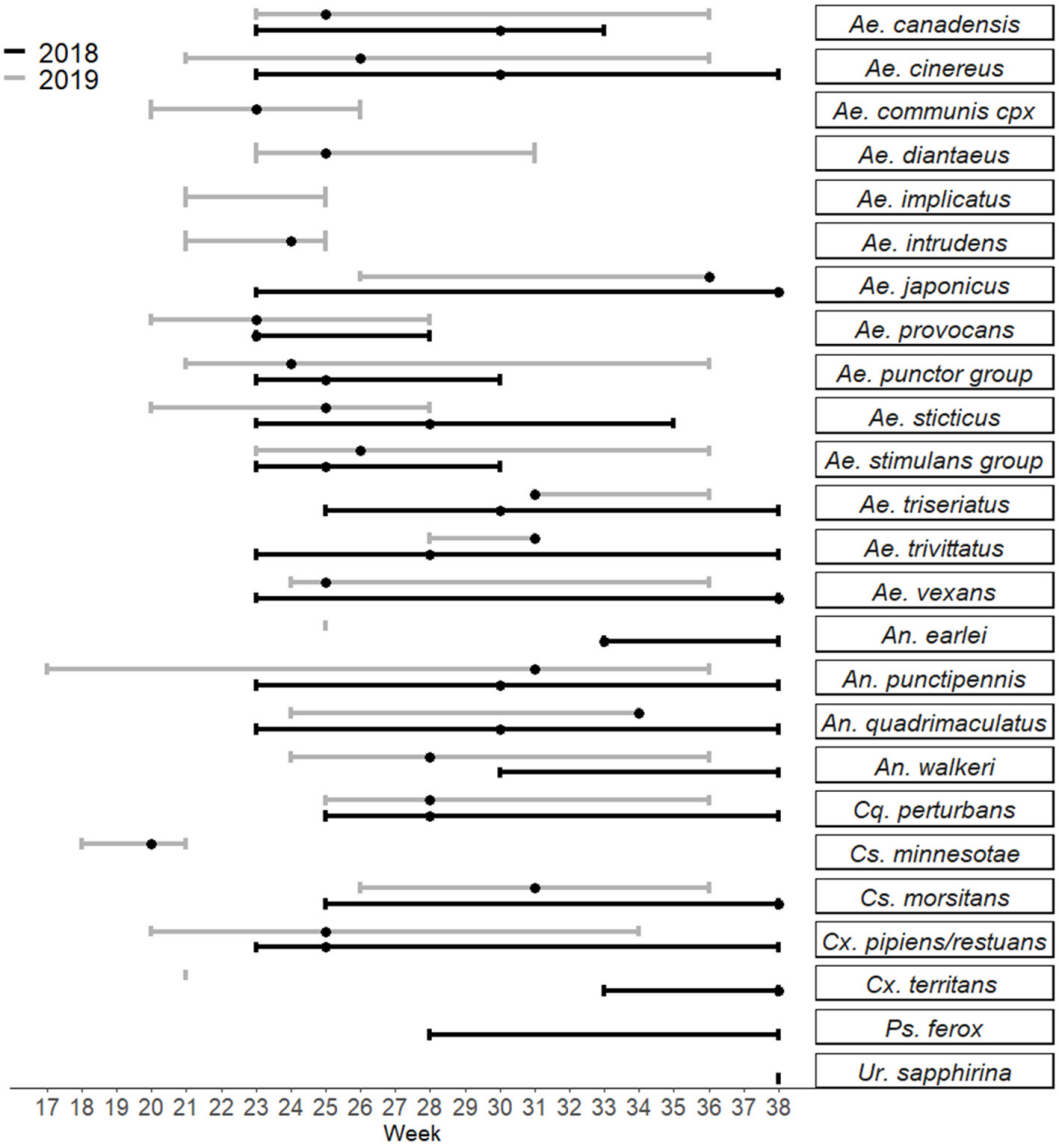
Seasonality of adult female mosquitoes trapped from 2018 to 2019 in Sawyer and Washburn Counties near Hayward, WI. Bars represent the range of weeks during which specimens were collected, and dots represent the week during which the species was most abundant in that time frame. Created in R using the package ggplot2 (25, 26).

Collections in Washburn and Sawyer Counties WI during 2018–2019 yielded specimens from 7 species/species complexes that are suspect vector species for Jamestown Canyon virus [(24); see Supplementary Table 2], including: *Aedes canadensis, Ae. communis* complex, *Ae. provocans, Ae. punctor* group, *Ae. stimulans* group, *Coquillettidia perturbans*, and *Anopheles punctipennis*. Normalized trap count data for these seven species are presented in Table 3. Here we provide brief details on total adult mosquitoes collected, the range of time during which specimens were collected, and detection of JCV. *Ae. canadensis*: In 2018, a total of 437 *Ae. canadensis* adult females were collected throughout the survey (weeks 23–33) (see Figure 1). A total of 4,881 adult females were collected in 2019 (weeks 23–36) (Figure 1). All of these mosquitoes were pooled and subjected to PCR testing for JCV, and no JCV was detected (Table 4). *Ae. communis* complex: No *Ae. communis* complex mosquitoes were collected in 2018. In 2019, a total of 36 adult females were collected. No JCV was detected in *Ae. communis* pools (Table 4). *Ae. provocans:* In 2018, a total of 60 adult female *Ae. provocans* were collected and tested; one pool (week 25) tested positive for JCV. In 2019, a total of 1,153 adult females were collected and tested during weeks 20–28 with peak abundance in week 23 (Figure 1). Four pools of adult female *Ae*. *provocans* collected in week 24, and one pool collected in week 26 tested positive for JCV. One pool of adult female *Ae*. *provocans*, collected during week 21 as immatures and reared to adult, tested positive for JCV (Table 4). *Ae. stimulans*: In 2018, a total of 29 *Ae. stimulans* females were collected and 1,500 adult females were collected in 2019. Jamestown Canyon virus was not detected in field-collected *Ae*. *stimulans* group mosquitoes (Table 4). *Cq. perturbans:* During 2018, a total of 13,782 female *Cq. perturbans* were collected during weeks 25–38. In 2019, a total of 3,373 adult females were collected during weeks 25–36 (Figure 1). We tested 10,698 of 13,782 mosquitoes in 2018 and did not detect JCV (Table 4). *An*. *punctipennis*: In 2018, a total of 366 *An*. *punctipennis* were collected and tested during weeks 23–38. In 2019, a total of 185 adult females were collected and tested during weeks 17–36 (Figure 1). JCV was not detected in this species (Table 4).

**Table 3:** Adult female mosquitoes collected in carbon dioxide-baited light traps, normalized as trap night (total collected/total trap events).

**Species**	**Adult females/Trap night 2018**	**Adult females/Trap night 2019**
*Ae. canadensis*	39.4	253.1
*Ae. communis* cpx	0	1.6
*Ae. provocans*	8.79	53.5
*Ae. punctor* group	14.1	120.4
*Ae. stimulans* group	3.5	77.6
*An. punctipennis*	20.7	6.3
*Cq. perturbans*	1312.6	267.6

*Traps were operated during weeks 23, 25, 28, 30, and 35 in 2018 and during weeks 23, 25, 28, 31, and 34 during 2019. Species listed are suspected vectors for JCV*.

**Table 4:** Total number of adult female mosquitoes collected in 2018 and 2019, Washburn and Sawyer Counties, Wisconsin, U.S.A., and pooled for JCV testing.

**Species**	**Total mosquitoes 2018**	**JCV+/Pools**	**Total mosquitoes 2019**	**JCV+/Pools**	**MFIR**
Ae. canadensis*	437	0/20	4,881	0/105	0:5,318
*Ae. cinereus*	260	0/19	4,855	0/112	0:5,115
*Ae. communis* cpx*	0	0	36	0/16	0:36
*Ae. diantaeus*	0	0	625	0/20	0:625
*Ae. implicatus*	0	0	3	0/3	0:3
*Ae. intrudens*	0	0	28	0/8	0:28
*Ae. japonicus*	59	0/7	3	0/2	0:62
Ae. provocans*	60	1/7	1,153	5/56	1:202
*Ae. punctor* group*	114	0/8	4,009	0/96	0:4,123
*Ae. sticticus*	431	0/26	1,867	0/47	0:2,298
*Ae. stimulans* group*	27	0/6	1,499	0/41	0:1,526
*Ae. triseriatus*	141	0/10	6	0/4	0:147
*Ae. trivittatus*	79	0/10	36	NT	0:79
*Ae. vexans*	826	0/33	116	NT	0:826
Cq. perturbans*	10,698	0/232	3,373	NT	0:10,698
*Cx. pipiens/restuans*	133	0/15	249	NT	0:133
*Cx. territans*	21	NT	2	NT	NT
*Cs. inornata*	8	0/1	12	0/6	0:20
*Cs. minnesotae*	0	0	11	0/7	0:11
*Cs. morsitans*	55	0/9	55	0/10	0:110
*An. earlei*	9	0/1	1	0/1	0:10
An. punctipennis*	366	0/23	185	0/19	0:551
*An. quadrimaculatus*	14	0/6	20	0/6	0:34
*An. walkeri*	10	0/5	28	0/6	0:38
*Ps. ferox*	2	NT	0	NT	NT
*Ur. sapphirina*	1	NT	0	NT	NT

Pools consisted of no more than 50 mosquitoes each. MFIR is a ratio of the total positive mosquito pools to the total number of mosquitoes tested for each species. Suspected vectors, denoted with^*^, are species that have met several criteria for vector incrimination. NT, not tested.

Collection efforts made in 2018 and 2019 also yielded several mosquito species that were tested for JCV but that are not clear suspect JCV vectors. These species included *Ae. cinereus, Ae. diantaeus, Ae. intrudens, Ae. implicatus, Ae. japonicus, Ae. sticticus, Ae. triseriatus, Ae. trivittatus, Ae. vexans, Culex pipiens, Cx. restuans, Cx. territans, Culiseta inornata, Cs. minnesotae, Cs. morsitans, Uranotaenia sapphirina, Psorophora ferox, Anopheles walkeri, An. quadrimaculatus*, and *An. earlei*. Here we provide brief details on total adult mosquitoes collected, the range of time during which specimens were collected, and detection of JCV. *Aedes cinereus:* In 2018, 261 *Ae. cinereus* females were collected. In 2019, a total of 4,856 (4,855 tested) adult females were collected. *Aedes diantaeus:* A total of 625 *Ae*. *diantaeus* females were collected during weeks 23–31 (Figure 1). *Aedes japonicus*: In 2018, 329 *Ae. japonicus* immatures were collected from tires. A total of 65 (59 tested) adult females were collected during weeks 23–38. In 2019, only 3 adult females were collected and tested between weeks 26 and 36 (Figure 1). *Aedes sticticus:* In 2018, a total of 442 (431 tested) *Ae. sticticus* females were collected. In 2019 a total of 1,867 adult females were collected and tested during weeks 20–28 (Figure 1). *Aedes triseriatus*: In 2018, 503 *Ae*. *triseriatus* immatures and 144 adult females were collected at a human JCV case site during weeks 25–38. *Aedes vexans:* In 2018, 868 adult *Ae*. *vexans* were collected (826 tested) during weeks 23–38. In 2019, a total of 116 adult females were collected during weeks 24–36. During 2018 and 2019, no JCV was detected in adult or immature collections from any of these species (Table 4). Collection and testing results for additional species of *Anopheles, Culex, Culiseta, Psorophora*, and *Uranotaenia* that were collected are detailed in Table 4.

## Discussion

In the search to implicate mosquitoes involved in transmission of a zoonotic arthropod-borne virus and human pathogen, a spectrum of mosquito behavioral and physiological attributes (termed “vectorial capacity”) is taken into account. The suspect vector must coincide in space and time with, and feed on the reservoir host. The suspect vector should be repeatedly recovered from the field with the virus. The presumed vector should acquire the virus upon feeding on an infectious blood meal, and transmission of the virus should be proven under controlled laboratory conditions (27). Furthermore, vectorial capacity to transmit the pathogen then depends on vector abundance, vector host preference and feeding behavior, vector competence and abiotic factors. Therefore, to best understand the public health risk and pathways to control for each species that could be involved in JCV transmission, we present each species collected according to its current taxonomic description, the presence or absence and abundance of immature and adult mosquitoes, the timing of collections (according to epidemiological week of the year) (Figure 1), JCV diagnostic information (Table 4), and the timing of collections and landscape features that provide habitat for immature mosquitoes (Tables 1, 2 and see Supplementary Figure 1). For context regarding the timing of mosquito collections and human cases, the case onset for JCV in Wisconsin can occur as early as April and through November, with peak case onset in July (5). The landscape of Sawyer County, WI provides ample habitat for immatures of mosquito species that are suspect JCV vectors because it is characteristically covered by glacial till over deeper bedrock with majority forested loamy and silty soils (28). The combination of compact till and silty soils contribute to the presence of a high density of snow melt and ephemeral pools that are breeding habitats for several species of mosquito potentially involved with JCV transmission (see Table 2).

### *Aedes (Ochlerotatus) canadensis* (Theobald, 1901)

*Aedes canadensis* is distributed throughout Canada as well as the Northern, Eastern, and Midwestern states in the U.S.A (29). This species does take blood meals from deer (28, 30–33). Jamestown Canyon virus was detected in 2 studies from field-collected adult female *Ae*. *canadensis* in Connecticut and Michigan, with a minimum field infection rate (MFIR) of 1:4,449 and 1:1,131 [(13, 34); Supplementary Table 2]. However, in other years in Connecticut and Michigan, and elsewhere (15, 18, 30, 34–37) (New York, Indiana, and Wisconsin), JCV was not detected in surveyed adult female *Ae*. *canadensis* (Supplementary Table 2), including this study (Table 4). Jamestown Canyon virus also was not detected in male nor immature *Ae*. *canadensis* collected from Connecticut (37), so there is no evidence to suggest its involvement in vertical transmission and transseasonal maintenance of JCV. In a laboratory study, *Ae*. *canadensis* was susceptible to JCV and transmitted virus, but the authors noted that the timing of emergence of this mosquito didn't align with human case occurrence (16).

This species is abundant in many areas of Wisconsin, and was frequently encountered in this study (5,836 specimens total). Specimens were collected throughout the summer from June to September (Figure 1). Based on historical MFIR data, we trapped and tested sufficient numbers (5,318 specimens) of *Ae*. *canadensis* to detect JCV (see Supplementary Table 2), but did not detect JCV. This result is congruent with mixed results on *Ae*. *canadensis* JCV infection status from other states.

### *Aedes (Ochlerotatus) communis* Complex

This well-defined species complex consists of *Aedes communis* (De Greer, 1776), *Aedes churchillensis* Ellis and Brust, 1973, *Aedes tahoensis* (Dyar, 1916), and *Aedes churchillensis* Ellis and Brust, 1973 (38). Adult females collected in this study were not identified beyond the level of the *Ae. communis* “aggregate” (39); however, thus far in WI only the nominotypical species is known (40), so it is probable that our specimens are *Ae*. *communis* s.s. This species is distributed throughout Canada and in the Western, upper Midwest, and Northeast U.S.A. (29). This species feeds on deer (31, 33). It is important here also to note that an informal taxonomic grouping of convenience by the name of the “*communis* group” [not to be confused with the well-characterized *communis* complex of Brust and Munstermann (38)] historically included *Ae*. *punctor, Ae*. *abserratus, Ae*. *sticticus*, and *Ae*. *provocans*, among many other Nearctic *Aedes (Ochlerotatus)* taxa. Defoliart et al. employed this broad concept in reference to JCV positive pools in WI; therefore, we do not know if these authors isolated JCV from *Ae*. *communis, Ae*. *punctor, Ae*. *abserratus, Ae*. *sticticus*, and/or *Ae*. *provocans* (36).

Jamestown Canyon virus has been detected in adult female *Ae*. *communis* in Connecticut (MFIR 1:612) and New York (MFIR 1:173), and in Wisconsin (MFIR 1:3,888, as “*Ae*. *communis* group”) [(13, 18, 36); Supplementary Table 2]. However, JCV was not found in mosquitoes collected from 1969 to 1978 in Connecticut (37), nor from 1981 to 1982 in Massachusetts [(41); Supplementary Table 2]. Jamestown Canyon virus was also not found in immature and male mosquitoes collected in New York and Connecticut [(18, 37); Supplementary Table 2] so is not likely to be involved in vertical transmission of JCV. To the best of our knowledge, *Ae*. *communis* has not been subjected to vector competence studies, and its vector status is suspect.

We collected a total of 77 *Ae*. *communis* complex adults, and noted greatest abundance in early June (week 23) (Figure 1). Immature *Ae. communis* complex specimens were exclusively collected in ephemeral pools (Table 2). We did not detect JCV in this species in this study. However, because we rarely encountered this species, we cannot draw conclusions about its bionomics and vector status for JCV.

### *Aedes (Hulecoeteomyia) japonicus* (Theobald, 1901)

*Ae*. *japonicus* was introduced into North America in the 1990's and rapidly invaded westward (42). This species was first reported in Wisconsin in Monroe county in 2004 (43), and was detected in 14 additional counties in 2016–2017 (44). Because the invasion of this species is more recent, the role of *Ae*. *japonicus* in JCV transmission is largely unknown. *Ae*. *japonicus* does have a propensity to feed on deer (28, 30, 33). JCV was not found in 509 adult female *Ae*. *japonicus* collected in Connecticut from 2010–2011 [(30); Supplementary Table 2]. This species has not been evaluated in laboratory studies for infection or transmission.

We collected a total of 413 specimens during the course of this study, and collections spanned from week 23 to 38. Notably, our data provide new species records for *Ae*. *japonicus* in 2 counties in Northern WI. Immature *Ae. japonicus* were exclusively collected in tires as compared to natural water sources (Table 2). We did not detect JCV in mosquitoes collected for this study (Table 4). However, we contend that the multivoltine nature of this species, its blood-feeding tendencies and vector competence for numerous arboviruses are strong support for additional lab and field studies to better understand its potential role in JCV transmission.

### *Aedes (Rusticoidus) provocans* (Walker, 1848)

This species is reported in western Canada along with southern Quebec and Ontario, and the northern third of the U.S.A. (29). *Ae*. *provocans* is a snowmelt mosquito that emerges from ephemeral pools with a population peak in the spring. *Ae*. *provocans* was noted as a “severe local pest” in parts of New York as well (31). Furthermore, this species is known to feed on deer (31). JCV-infected adult female *Ae*. *provocans* have been detected in Connecticut, New York, and Michigan (13, 18, 34). Elsewhere, JCV was not found in adult females, including Connecticut, and Massachusetts [(37, 41); Supplementary Table 2]. In a laboratory setting, this species is susceptible to JCV and 20–50% of the infected individuals develop disseminated infection with JCV (16). Boromisa and Grayson confirmed that *Ae*. *provocans* transmits JCV in controlled laboratory settings (14). Additionally, there is some evidence that *Ae*. *provocans* maintains JCV over the winter because JCV was found in adults collected as immatures from New York (18), but not from *Ae*. *provocans* collected in Connecticut [(37); Supplementary Table 2].

In this study, we collected 2,799 *Ae*. *provocans*, and noted peak adult activity in early June (week 23) (Figure 1). The majority of *Ae. provocans* collected as immatures in this study came from ephemeral pools (see Table 2 and Supplementary Figure 1). This species can tolerate extreme cold temperatures as those ephemeral pools were still covered with snow and ice at the time of collection. The timing and emergence of *Ae. provocans* are linked with very early spring nectar sources (45). We noted that flowering *Anemone* spp. coincide with *Ae*. *provocans* emergence in Sawyer County WI. In Ontario, *Ae*. *provocans* display a synchronous emergence with 95% of the population emerging over the course of 11–14 days, which can accelerate if appropriate nectar sources are present (46). Adult female *Ae*. *provocans* collected in week 25 in 2018, and weeks 24 and 26 in 2019, tested positive for JCV (Table 1). A pool of adult females collected as immatures during week 21 also tested positive, providing further evidence for vertical transmission (Table 1). This is the first report of a JCV positive female *Ae*. *provocans* collected during an immature stage. Repeat detection of JCV in field-collected adult mosquitoes over 2 years, and detection from mosquitoes collected as juveniles point to the importance of *Ae*. *provocans* in vertical and early season transmission dynamics of JCV in northern parts of the Upper Midwest.

### *Aedes (Ochlerotatus) punctor* Group

This informal species grouping, as employed by some authors [see e.g., Steward, 1968; (46)], includes *Aedes aboriginis* Dyar, 1917, *Aedes hexodontus* Dyar, 1916, *Aedes punctor* (Kirby, 1837), *Aedes punctodes* Dyar, 1922, and *Aedes abserratus* (Felt and Young, 1904), with only *Ae*. *punctor* and *Ae*. *abserratus* being recorded in Wisconsin (40). This species-grouping was used here due to the difficulty of identifying these species based on adult female morphology alone, but we conclude that specimens collected in this study were *Ae*. *punctor* and/or *Ae*. *abserratus*. *Ae*. *abserratus* has been reported in eastern Canada as well as the upper Midwest and Northeast U.S.A. (29). Furthermore, although we could not identify adult female specimens definitively, we did note that only *Ae*. *punctor* larvae were observed in the samples collected in 2019. This species has been found to feed on deer (47). Adult female, JCV-positive *Ae*. *punctor* group have been detected with JCV from Connecticut, New York, Michigan, and Massachusetts [(13, 18, 34, 37, 41); Supplementary Table 2]. Jamestown Canyon virus was not detected in adult females of this species during surveillance in Connecticut [(30); Supplementary Table 2]. JCV also was not found in immatures nor males collected from New York and Connecticut [(18, 37); Supplementary Table 2]. Interestingly, species in this group can support disseminated JCV infection, but in a controlled lab setting did not transmit virus (14), so virus detections from this group may reflect only evidence of having blood fed on a viremic host, and not transmission potential.

In this study, we collected 4,009 *Aedes punctor* group specimens with adult abundance peak at week 24 (Figure 1). Based on previously published MFIR, this should have been sufficient to detect JCV (Supplementary Table 2). Immatures were collected in ephemeral pools, permanent water bodies and small ponds (Table 4). We did not detect JCV in specimens of this species in this study (Table 2).

### *Aedes (Ochlerotatus) stimulans* Group

This is another informal grouping of convenience, utilized by some authors as including *Aedes stimulans* s.s. (Walker, 1848), *Aedes fitchii* (Felt and Young, 1904), *Aedes flavescens* (Müller, 1764), *Aedes euedes* Howard, Dyar and Knab 1913, 1917. *Aedes excrucians* (Walker, 1856), *Aedes riparius* Dyar and Knab, 1907, *Aedes aloponotum* (Dyar, 1917), *Aedes grossbecki* (Dyar and Knab, 1906), *Aedes increpitus* Dyar, 1916, *Aedes clivis* Lanzaro and Eldridge, 1992, *Aedes washinoi* Lanzaro and Eldridge, 1992, *Aedes mercurator* Dyar, 1920, and *Aedes dahlae* (Nielsen, 2009) (48–50). The first six of these have been recorded in Wisconsin (40). Remarkably few of these taxa are readily separable in the adult female, and thus our identification stops short of species-level. We identified at least *Ae*. *excrucians* and *Ae*. *fitchii* in the larval stage, in a small subset of larval collections that was identified prior to adult emergence, but it is unknown which, if any, additional *Ae*. *stimulans* group taxa may have been collected. We consider it likely that it was some combination of the 6 species previously collected by Gilardi and Hilsenhoff (40).

*Ae*. *stimulans* has been reported in southern Ontario and Quebec, as well as in the Midwest and Northeast U.S.A. (29). *Ae*. *fitchii* has been reported in Canada and the northern half of the U.S.A. (29). *Ae*. *excrucians* has been reported in Canada and the northern third of the U.S.A. (29). *Ae*. *euedes* is reported as having a patchy distribution, represented by isolated pockets in Alaska, western Canada, pockets in the Rocky Mountains of the U.S., broadly across central Canada and the north-central U.S., and a patch in New Brunswick, Canada (29). *Ae*. *flavescens* is recorded as occurring broadly across the central third of the Nearctic region, with an isolated patch in Newfoundland (29). *Aedes riparius* occurs in inland areas of Alaska, Canada, and the northernmost central U.S. states, as well as near coastal New Brunswick (29).

Members of this group do feed on deer (30–33, 47). Additionally, adult female *Ae*. *stimulans* group have been found infected with JCV in Connecticut, Indiana, and Wisconsin [(13, 15, 30, 36); Supplementary Table 2]. Jamestown Canyon virus was not found in adult female mosquitoes during surveillance in New York, Michigan, and Connecticut [(18, 34, 37); Supplementary Table 2]. JCV was found in male and immature *Ae*. *stimulans* group collections from Indiana (15), but not in collections from New York and Connecticut [(18, 37); Supplementary Table 2], so this mosquito may play a role in transseasonal transmission of JCV. *Ae*. *stimulans* s.s. is a competent vector in laboratory studies, where it displayed disseminated infection and transmission (15, 16).

We collected 2,041 *Ae*. *stimulans* group during the study period and observed peak adult abundance in weeks 25–26 (Figure 1). Immatures were collected in ephemeral pools, permanent water bodies, and small ponds (Table 2). No *Ae*. *stimulans* group tested positive for JCV in this study (Table 4). Overall, our results do not rule out the possibility that *Ae*. *stimulans* group mosquitoes are part of the JCV transmission cycle in Wisconsin.

### *Coquillettidia (Coquillettidia) perturbans* (Walker, 1856)

This species is distributed across southern Canada and the majority of the U.S.A. (19). This species has been found to feed on deer (28, 30, 32, 33). Adult female *Cq*. *perturbans* have been found infected with JCV in Connecticut [(13, 30, 37); Supplementary Table 2]. Jamestown Canyon virus was not found in adult female mosquitoes during surveillance in North Dakota, New York, Indiana, Wisconsin, and Michigan, despite large numbers of mosquitoes tested [(15, 18, 34, 51); Supplementary Table 2]. This virus also was not found in male and immature collections from Connecticut, so is not likely to transmit this virus vertically (Supplementary Table 2). When subjected to laboratory infection, this species did develop disseminated infection and transmitted virus (16).

In this study, we collected a total of 17,155 (13,782 in 2018, 3,373 in 2019) *Cq*. *perturbans*, and noted peak adult abundance in week 28 in both years. We tested a portion of these (>10,000) in 2018, and did not detect JCV; therefore, we elected to not use resources to test these mosquitoes in 2019. Because this is a very abundant species, with a low MFIR for JCV [e.g., 1:23,429 and 1:26,666 in mosquitoes from CT (13, 37)], we recommend careful consideration of cost:benefit when making decisions to invest in identification, pooling, and testing *Cq. perturbans* specimens for this virus.

### *Anopheles (Anopheles) punctipennis* (Say, 1823)

This species is distributed across southern Canada and throughout the U.S.A. (29). It has been known to feed on deer (28, 30, 33, 52). Adult female *An*. *punctipennis* have been found infected with JCV in Connecticut [(13); Supplementary Table 2]. Jamestown Canyon virus was not found in adult female mosquitoes during other years of surveillance in Connecticut, or New York, Indiana, and Michigan [(15, 18, 30, 34, 37); Supplementary Table 2]. Jamestown Canyon virus was also not found in male and immature collections from Connecticut [(37); Supplementary Table 2]. This species is a competent vector according to laboratory studies (16).

We collected 556 *An*. *punctipennis* during this study and noted that adults were present throughout the study period during both years, with peak abundance in week 30–31 (late July). We did not detect JCV in specimens collected during this study (Table 4). However, in the study where JCV was detected in this species, the MFIR was 1:1,457 (13), so we may not have collected sufficient numbers of species to detect JCV. Given its vector competence, propensity to feed on deer, multivoltine reproduction and representation for the duration of the season (see Figure 1), this species warrants additional study for its role in JCV transmission in WI.

## Summary

Based on historical mosquito surveillance data, serosurveillance in deer, epidemiological week range of human case onset, and the data presented from this study, we contend that JCV is maintained in Wisconsin as it is elsewhere, in both vertical and horizontal transmission cycles. Epidemic levels of JCV-associated disease in Wisconsin inspired an intensive, 2 year surveillance effort on the Lac Courte Oreilles Reservation and in Sawyer and Washburn Counties in Wisconsin to understand the infection status and ecology of suspect JCV vector species. Based on extensive review of the literature (see Supplementary Table 2), and on the mosquito fauna described for Wisconsin, we suspected that the most likely vectors for JCV in this area include *Ae*. *canadensis, Ae*. *communis* complex*, Ae*. *provocans, Ae*. *punctor* group, *Ae*. *stimulans* group, and *An*. *punctipennis*. There are as many as 53 species of mosquito reported in the state (53), and we collected 26 species or groups of species during the 2018 and 2019 field seasons at these northern Wisconsin sites, including the 7 suspect species/species group mosquitoes. Despite extensive sampling, only one of the suspect species, *Ae*. *provocans*, consistently tested positive for JCV by PCR; importantly, material from these pools also yielded virus growth in cell culture, thereby confirming that these collections harbored mosquitoes with infectious virus. JCV was found in both 2018 and 2019 in *Ae*. *provocans*; 6 positive pools came from a single site, one from 2018, and 5 from 2019. Additionally, one pool of *Ae*. *provocans*, collected as immatures in week 21 in 2019, tested positive for JCV and provided evidence that this species serves as an important transseasonal maintenance host for the virus.

There is much yet to be learned about the full spectrum of vector and reservoir hosts involved in the JCV transmission cycle in Wisconsin and elsewhere. Human JCV cases in Wisconsin are reported throughout the entire summer through fall, with peak incidence in July through August (4). Ungulates, and deer in particular in the Midwest, are well-understood as the primary reservoir host of JCV (8, 9, 54). Historical serosurveys of deer in Wisconsin indicate that 20–90% of deer have been exposed to JCV (8). Cohort serial testing of deer populations in Michigan indicate that fawns are seropositive to JCV as newborns (indicative of maternal antibody), become seronegative in the fall through winter, and seroconvert in the spring as yearlings (8). All yearling deer in one study had seroconverted to JCV by the end of June (10). Artificial JCV inoculation of deer in a laboratory setting indicate deer are only viremic for about four days following infection (8). Therefore, the window during which deer may serve as a source of JCV to a vector is very short, and not likely to extend beyond July (at least in areas with endemic JCV and competent and infected early-season *Aedes* spp.). Early-emerging spring *Aedes* mosquitoes in Sawyer County significantly decrease in abundance between June and July and therefore are not likely to contribute to human transmission in July and August (see Figure 1). It is possible that (1) additional vertebrate hosts serve as reservoirs for the virus, and/or (2) a secondary and long-lived vector, such as *Ae*. *canadensis* or *An*. *punctipennis*, acquires infection in June and transmits virus to humans for the duration of the summer.

The results from this study are a benchmark for future work on vector and non-vector species composition and abundance in an epidemiological “hot spot” for JCV, and for studies on vector competence and transmission dynamics. This work also highlights the seasonality and habitat preference for *Ae. provocans*—a species from which we repeatedly detected JCV—should there be interest in devising control strategies to prevent transmission in the future.

## Data Availability Statement

The original contributions presented in the study are included in the article/Supplementary Material, further inquiries can be directed to the corresponding author.

## Author Contributions

MF, NT, BT, and LB: study design. MF, NT, and BT: study execution. BT and LB: supervision. LB: resources and funding. MF: manuscript draft. BT and MF: figure preparation. All authors contributed to manuscript revision, read, and approved the submitted version.

## Funding

This publication was supported by Cooperative Agreement #U01CK000505, funded by the Centers for Disease Control and Prevention.

## Land Grant Acknowledgment

The University of Wisconsin–Madison occupies ancestral Ho-Chunk land, a place their nation has called Teejop (day-JOPE) since time immemorial. In an 1832 treaty, the Ho-Chunk were forced to cede this territory. Decades of ethnic cleansing followed when both the federal and state government repeatedly, but unsuccessfully, sought to forcibly remove the Ho-Chunk from Wisconsin. This history of colonization informs our shared future of collaboration and innovation. Today, UW–Madison respects the inherent sovereignty of the Ho-Chunk Nation, along with the 11 other First Nations ofWisconsin.

## Author Disclaimer

Its contents are solely the responsibility of the authors and do not necessarily represent the official views of the Centers of Disease Control and Prevention or the Department of Health and Human Services.

## Conflict of Interest

The authors declare that the research was conducted in the absence of any commercial or financial relationships that could be construed as a potential conflict of interest.

## Publisher's Note

All claims expressed in this article are solely those of the authors and do not necessarily represent those of their affiliated organizations, or those of the publisher, the editors and the reviewers. Any product that may be evaluated in this article, or claim that may be made by its manufacturer, is not guaranteed or endorsed by the publisher.
