# Supplementary material for: Outbreak Investigation: Jamestown Canyon Virus Surveillance in Field-Collected Mosquitoes (Diptera: Culicidae) From Wisconsin, USA, 2018–2019
Source: Front Public Health. 2022 Apr 21;10:818204. doi: 10.3389/fpubh.2022.818204 (PMC9068969; doi:10.3389/fpubh.2022.818204)
Supplement: Supplementary file 1 [file Image_1.pdf]

Supplemental Figure 1. Location and habitat examples for immature and adult mosquito collections made near Hayward, Wisconsin, U.S.A. during 2018 and 2019. The figure map below was produced using QGIS (55).

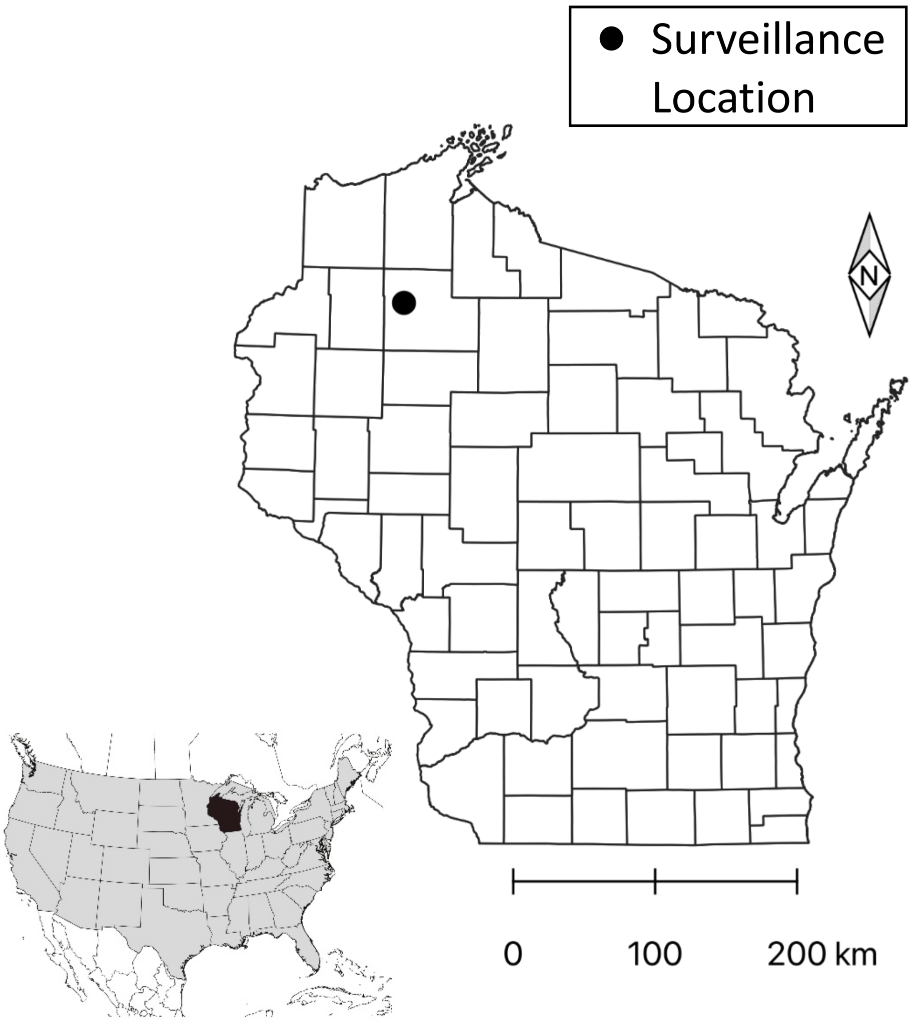

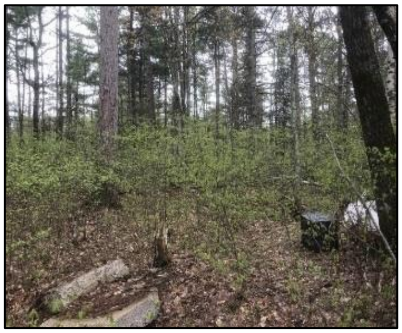

Adult mosquitoes were collected using CDC light, BG Sentinel traps, and resting boxes in this forest habitat.

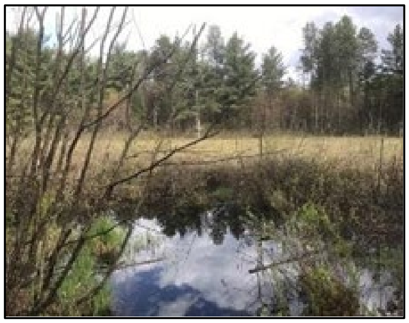

Immature and adult mosquitoes were collected from permanent marsh habitat.

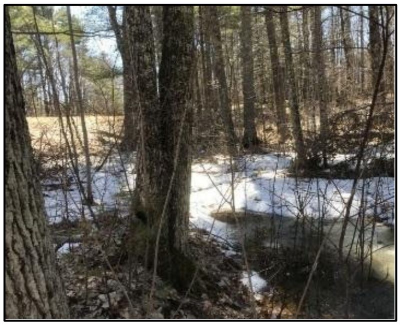

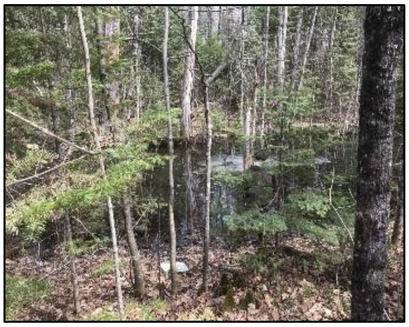

Immature and adult mosquitoes were collected from these ephemeral pools.
