# Supplementary material for: Outbreak Investigation: Jamestown Canyon Virus Surveillance in Field-Collected Mosquitoes (Diptera: Culicidae) From Wisconsin, USA, 2018–2019
Source: Front Public Health. 2022 Apr 21;10:818204. doi: 10.3389/fpubh.2022.818204 (PMC9068969; doi:10.3389/fpubh.2022.818204)
Supplement: Supplementary file 2 [file Image_2.pdf]

Supplemental Figure 2. Methods used to collect immature and adult mosquitoes in 2018 and 2019.

Carbon dioxide baited CDC miniature light trap (John W. Hock Company)	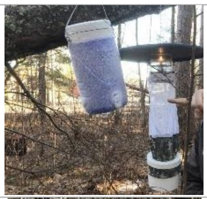	The carbon dioxide baited CDC miniature light trap was used to collect host-seeking adult mosquitoes from April-September, 2018/2019.
Resting Box (Silver 2008)	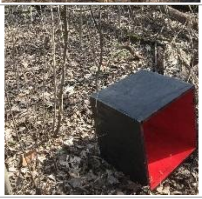	The resting box collection method targets resting adult males and females and was used in April and May, 2019.
Carbon dioxide baited BG-Sentinel 2 (Biogents)	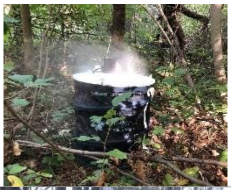	The carbon dioxide baited BG Sentinel 2 was used to collect adult host-seeking mosquitoes from June-September, 2019.
Shannon Trap Baited with UV Light (Silver 2008)	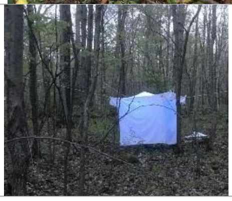	The Shannon trap is baited with UV-light and used to attract adult where they are manually collected. This method targets both adult males and females and was used in May, 2019.
Modified CDC backpack aspirator (John W. Hock Company)	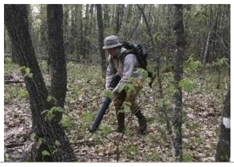	This method targets resting adult male and female mosquitoes by vacuuming them from vegetation and was used in May, 2019.
Bioquip mosquito dipper (Bioquip)	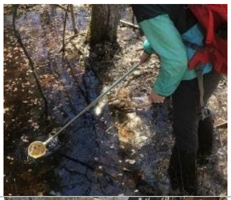	This is the standard method that was used to collect immature mosquitoes from all water sources in April-May, 2019 and May-September, 2018.
Aquatic Light Trap (Bioquip)	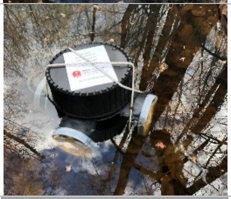	The aquatic light trap was used to collect immature mosquitoes from ephemeral pools. This method was utilized in April and May, 2019.
Emergence Trap (Silver 2008)	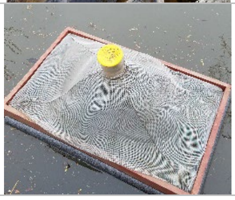	The emergence trap was used as it bypassed the need to raise the immature stages to adults in the lab post-collection. This method was used in May, 2019.
