# Supplementary material for: Outbreak Investigation: Jamestown Canyon Virus Surveillance in Field-Collected Mosquitoes (Diptera: Culicidae) From Wisconsin, USA, 2018–2019
Source: Front Public Health. 2022 Apr 21;10:818204. doi: 10.3389/fpubh.2022.818204 (PMC9068969; doi:10.3389/fpubh.2022.818204)
Supplement: Supplementary file 3 [file Table_1.docx]

Supplemental Table 1. Precipitation and temperature data for 2018-1019, Sawyer County, Wisconsin during epidemiological weeks 1-40. A) precipitation data for weeks 1-40 as compared to National Oceanic and Atmospheric Administration (NOAA) 30 year normal values and B) Temperature data and deviation from 30 year normal for epidemiological weeks 16-38. Weather data were obtained from NOAA (56).

A.

| **Year** | **Precipitation Weeks 1-20 (mm)** | **Precipitation Weeks 21-40 (mm)** |
| --- | --- | --- |
| **2018** | 72.64 | 685.038 |
| **2019** | 278.89 | 600.71 |
| **NOAA 1981-2010 30 year normal** | **19.89** | **43.13** |

B.

| **Weeks** | **Temperature (celcius) 2018** | **Deviation from normal 2018** | **Temperature (celcius) 2019** | **Deviation from normal 2019** | **NOAA 1981-2010 normal (celcius)** |
| --- | --- | --- | --- | --- | --- |
| **16** | 0.08 | -5.88 | 5.71 | -0.24 | 5.96 |
| **17** | 5.40 | -2.25 | 8.17 | 0.53 | 7.64 |
| **18** | 13.49 | 4.29 | 5.87 | -3.33 | 9.20 |
| **19** | 11.75 | 1.17 | 6.75 | -3.83 | 10.57 |
| **20** | 14.68 | 2.85 | 10.71 | -1.11 | 11.83 |
| **21** | 18.17 | 5.19 | 10.40 | -2.59 | 12.99 |
| **22** | 19.05 | 4.95 | 14.29 | 0.19 | 14.10 |
| **23** | 15.16 | -0.04 | 16.90 | 1.70 | 15.20 |
| **24** | 20.00 | 3.73 | 13.49 | -2.78 | 16.27 |
| **25** | 20.48 | 3.18 | 16.67 | -0.63 | 17.30 |
| **26** | 21.98 | 3.83 | 18.89 | 0.73 | 18.16 |
| **27** | 20.56 | 1.76 | 21.51 | 2.71 | 18.80 |
| **28** | 22.46 | 3.26 | 20.48 | 1.28 | 19.20 |
| **29** | 19.44 | 0.10 | 22.94 | 3.59 | 19.34 |
| **30** | 17.70 | -1.60 | 19.52 | 0.22 | 19.30 |
| **31** | 17.54 | -1.62 | 19.21 | 0.05 | 19.16 |
| **32** | 20.71 | 1.89 | 18.57 | -0.26 | 18.83 |
| **33** | 21.19 | 2.82 | 19.13 | 0.76 | 18.37 |
| **34** | 18.02 | 0.34 | 15.56 | -2.12 | 17.67 |
| **35** | 18.41 | 1.70 | 15.40 | -1.32 | 16.71 |
| **36** | 16.35 | 0.89 | 14.63 | -0.83 | 15.46 |
| **37** | 19.92 | 5.98 | 13.73 | -0.21 | 13.94 |
| **38** | 14.44 | 2.13 | 20.24 | 7.92 | 12.31 |
| **Weeks 16-26** |  | **21.02** |  | **-11.36** |  |
| **Weeks 27-38** |  | **17.65** |  | **11.79** |  |
