# Supplementary material for: Outbreak Investigation: Jamestown Canyon Virus Surveillance in Field-Collected Mosquitoes (Diptera: Culicidae) From Wisconsin, USA, 2018–2019
Source: Front Public Health. 2022 Apr 21;10:818204. doi: 10.3389/fpubh.2022.818204 (PMC9068969; doi:10.3389/fpubh.2022.818204)
Supplement: Supplementary file 4 [file Table_2.docx]

**Supplemental Table 2**: Historical Minimum Field Infection Rates (MFIR) for mosquito species collected and infected with Jamestown Canyon virus from states in the U.S.A. MFIR is a ratio of the total positive mosquito pools to the total number of mosquitoes tested for each species, as reported. Refer to main text for reference numbers (#).

| **Reference (#)** | **MFIR *Aedes abserratus/Ae. punctor* group** | **YEAR/LOCATION** |
| --- | --- | --- |
| (27) | 0:11 | **Years :** 2010-2011  **Location :** Connecticut |
| (13) | 1:1,195 | **Years :** 1997-2006  **Location :** Connecticut |
| (18) | *Ae. abserratus*: 0:571  *Ae. punctor*: 1:727  Reared adults: 0:80 | **Years :** 1988-1989  **Location :** New York |
| (34) | 1:3,888  “*Ae*. *communis* grp” made up of *Ae. communis, Ae. punctor, Ae. abserratus, Ae. sticticus, Ae. provocans* (as *Ae. trichurus*) | **Years :** 1965  **Location :** Wisconsin |
| (25) | 1:382 | Review paper, 1990  **Years :** NA |
| (32) | 1984: 0:228  1985: 0:1,112  1987: 0:21  1988: 2:896 (1:448)  1989: 0:2,613  Total: 1:2,435 | **Years :** 1984-1989  **Location :** Michigan |
| (35) | 1:106 to 1:700 depending on year;  1:438 for all years combined  Males and immatures: 0:260 | **Years :** 1969-1978  **Location :** Connecticut |
| (42) | 1:3,844 | **Years :** 1981-1982  **Location :** Massachusetts |

| **Reference** | **MFIR *Ae. canadensis*** | **YEAR/LOCATION** |
| --- | --- | --- |
| (27) | 0:684 | **Years :** 2010-2011  **Location :** Connecticut |
| (13) | 1:4,449 | **Years :** 1997-2006  **Location :** Connecticut |
| (18) | 0:2,146  Males: 0:332  Reared adults: 0:18 | **Years :** 1988-1989  **Location :** New York |
| (15) | 0:152 | **Years :** 1982-1984  **Location :** Indiana |
| (34) | 0:1,998 | **Years :** 1965  **Location :** Wisconsin |
| (32) | 1984: 0:1,060  1985: 0:1,202  1987: 0:16  1988: 1: 1,131  1989: 0:1  Total: 1: 3,410 | **Years :** 1984-1989  **Location :** Michigan |
| (35) | 0:22,392  Males and immatures: 0:2,241 | **Years :** 1969-1978  **Location :** Connecticut |
| (42) | 0:1,222 | **Years :** 1981-1982  **Location :** Massachusetts |

| **Reference** | **MFIR *Ae. cinereus*** | **YEAR/LOCATION** |
| --- | --- | --- |
| (38) | 0:6,678 | **Years :** 2003-2006  **Location :** North Dakota |
| (27) | 0:1,299 | **Years :** 2010-2011  **Location :** Connecticut |
| (13) | 1:11,961 | **Years :** 1997-2006  **Location :** Connecticut |
| (18) | 0:1,007  Reared adults: 0:2 | **Years :** 1988-1989  **Location :** New York |
| (15) | 0:399 | **Years :** 1982-1984  **Location :** Indiana |
| (34) | 0:2,911 | **Years :** 1965  **Location :** Wisconsin |
| (25) | 1:2,647 | Review paper, 1990 |
| (32) | 0:4,274 | **Years :** 1984-1989  **Location :** Michigan |
| (35) | 0:5,344  Males and immatures: 0:2,160 | **Years :** 1969-1978  **Location :** Connecticut |
| (42) | 0:151 | **Years :** 1981-1982  **Location :** Massachusetts |

| **Reference** | **MFIR *Ae. communis* complex** | **YEAR/LOCATION** |
| --- | --- | --- |
| (13) | 1:612 | **Years :** 1997-2006  **Location :** Connecticut |
| (18) | 1989 – 1:173  Males in “*Ae*. *communis* group” – 0:1,240  Reared adults in “*Ae*. *communis* group” – 0:385 | **Years :** 1988-1989  **Location :** New York |
| (34) | 1:3,888  “*Ae*. *communis* grp” made up of *Ae. communis, Ae. punctor, Ae. abserratus, Ae. sticticus, Ae. provocans* (as *Ae. trichurus*) | **Years :** 1965  **Location :** Wisconsin |
| (25) | 1:1,538 (reported as “*Ae*. *communis* group”) | Review paper, 1990 |
| (35) | 0:15  Males and immatures: 0:11 | **Years :** 1969-1978  **Location :** Connecticut |
| (42) | 0:6,001 | **Years :** 1981-1982  **Location :** Massachusetts |

| **Reference** | **MFIR *Ae. diantaeus*** | **YEAR/LOCATION** |
| --- | --- | --- |
| (18) | 0:829 | **Years :** 1988-1989  **Location :** New York |
| (34) | Not reported; possibly a part of “*Ae*. *communis* group” | **Years :** 1965  **Location :** Wisconsin |
| (42) | 0:370 *Ae. dianteus, Ae. decticus* | **Years :** 1981-1982  **Location :** Massachusetts |

| **Reference** | **MFIR *Ae. implicatus*** | **YEAR/LOCATION** |
| --- | --- | --- |
| (34) | Not reported, possibly a part of “*Ae*. *communis* group” | **Years :** 1965  **Location :** Wisconsin |
| (32) | 0:12 | **Years :** 1984-1989  **Location :** Michigan |
| (42) | 0:3 | **Years :** 1981-1982  **Location :** Massachusetts |

| **Reference** | **MFIR *Ae. intrudens*** | **YEAR/LOCATION** |
| --- | --- | --- |
| (18) | 1:1,911  Reared adults: 0:174 | **Years :** 1988-1989  **Location :** New York |
| (34) | Not reported, possibly a part of “*Ae*. *communis* group” | **Years :** 1965  **Location :** Wisconsin |
| (32) | 1984: 0:40  1985: 1:82  1987: 0:1  1988: 0:121  1989: 0:96  Total: 1:211 | **Years :** 1984-1989  **Location :** Michigan |
| (35) | 0:9 | **Years :** 1969-1978  **Location :** Connecticut |
| (42) | 1:1,530 | **Years :** 1981-1982  **Location :** Massachusetts |

| **Reference** | **MFIR *Ae. japonicus*** | **YEAR/LOCATION** |
| --- | --- | --- |
| (27) | 0:509 | **Years :** 2010-2011  **Location :** Connecticut |

| **Reference** | **MFIR *Ae. provocans*** | **YEAR/LOCATION** |
| --- | --- | --- |
| (13) | 1:226 | **Years :** 1997-2006  **Location :** Connecticut |
| (18) | 1988 - 1:219  1989 - 1:429  1989 : Reared adults - 1:2,110 | **Years :** 1988-1989  **Location :** New York |
| (34) | 1:3,888  “*Ae*. *communis* grp” made up of *Ae. communis, Ae. punctor, Ae. abserratus, Ae. sticticus, Ae. provocans* (as *Ae. trichurus*) | **Years :** 1965  **Location :** Wisconsin |
| (25) | 1:495 | Review paper, 1990 |
| (32) | 1984: 0:58  1985: 1:112  1987: 1:27  1988: 1:320  1989: 1:714  Total: 1:499 | **Years :** 1984-1989  **Location :** Michigan |
| (35) | 0:1  Males and immatures: 0:12 | **Years :** 1969-1978  **Location :** Connecticut |
| (42) | 0:2,497 | **Years :** 1981-1982  **Location :** Massachusetts |

| **Reference** | **MFIR *Ae. sticticus*** | **YEAR/LOCATION** |
| --- | --- | --- |
| (38) | 1:34,132 | **Years :** 2003-2006  **Location :** North Dakota |
| (27) | 0:256 | **Years :** 2010-2011  **Location :** Connecticut |
| (13) | 1:4,402 | **Years :** 1997-2006  **Location :** Connecticut |
| (18) | 0:211 | **Years :** 1988-1989  **Location :** New York |
| (15) | 0:4,613 | **Years :** 1982-1984  **Location :** Indiana |
| (34) | 1:3,888  “*Ae*. *communis* grp” made up of *Ae. communis, Ae. punctor, Ae. abserratus, Ae. sticticus, Ae. provocans* (as *Ae. trichurus*) | **Years :** 1965  **Location :** Wisconsin |
| (32) | 0:1 | **Years :** 1984-1989  **Location :** Michigan |
| (35) | 0:8 | **Years :** 1969-1978  **Location :** Connecticut |

| **Reference** | **MFIR *Ae. stimulans* group** | **YEAR/LOCATION** |
| --- | --- | --- |
| (27) | *Ae. excrucians* – 1:53 | **Years :** 2010-2011  **Location :** Connecticut |
| (13) | 1:1,998 (*Ae. stimulans* combined with *Ae. excrucians*)  *Ae. stimulans* - 1: 4,719  *Ae. excrucians* – 1:1,092 | **Years :** 1997-2006  **Location :** Connecticut |
| (18) | 0:2,117  Males: 0:2,044  Reared adults: 0:106 | **Years :** 1988-1989  **Location :** New York |
| (15) | 1:1,260 in 1983.  May (1:591).  Males, May (1:1,424)  1:2,949 overall | **Years :** 1982-1984  **Location :** Indiana |
| (34) | 1:1,300  *Ae. excrucians, Ae. fitchii, Ae. stimulans* | **Years :** 1965  **Location :** Wisconsin |
| (25) | 1:2,682 (as *Ae*. *stimulans* s.s.)  1:4,446 (as *Ae*. *stimulans* group) | Review paper, 1990 |
| (32) | 0:1,826  *Ae*. *excrucians* and *Ae*. *fitchii* together | **Years :** 1984-1989  **Location :** Michigan |
| (35) | 0:3,023  Males and immatures: 0:277  *Ae. excrucians, Ae. fitchii, Ae. stimulans* | **Years :** 1969-1978  **Location :** Connecticut |
| (42) | 0:2,264 (as *Ae*. *stimulans* group) | **Years :** 1981-1982  **Location :** Massachusetts |

| **Reference** | **MFIR *Ae. triseriatus*** | **YEAR/LOCATION** |
| --- | --- | --- |
| (38) | 0:257 | **Years :** 2003-2006  **Location :** North Dakota |
| (27) | 0:51 | **Years :** 2010-2011  **Location :** Connecticut |
| (13) | 1:10,506 | **Years :** 1997-2006  **Location :** Connecticut |
| (17) | 1:676 reared larvae | **Years:** 7/21 – 8/12 1975  **Location:** Ohio |
| (18) | 0:3 | **Years :** 1988-1989  **Location :** New York |
| (15) | 0:233 | **Years :** 1982-1984  **Location :** Indiana |
| (34) | 0:174 | **Years :** 1965  **Location :** Wisconsin |
| (32) | 0:21 | **Years :** 1984-1989  **Location :** Michigan |
| (35) | 0:4,877  Males and immatures: 0:8,521 | **Years :** 1969-1978  **Location :** Connecticut |
| (42) | 0:1,552 adults  0:22,557 larvae | **Years :** 1981-1982  **Location :** Massachusetts |

| **Reference** | **MFIR *Ae. trivittatus*** | **YEAR/LOCATION** |
| --- | --- | --- |
| (38) | 1:21,629 | **Years :** 2003-2006  **Location :** North Dakota |
| (27) | 1:3,694 | **Years :** 2010-2011  **Location :** Connecticut |
| (13) | 1:19,827 | **Years :** 1997-2006  **Location :** Connecticut |
| (18) | 0:16 | **Years :** 1988-1989  **Location :** New York |
| (15) | 0:9,159 | **Years :** 1982-1984  **Location :** Indiana |
| (34) | 0:3,253 | **Years :** 1965  **Location :** Wisconsin |
| (32) | 0:1 | **Years :** 1984-1989  **Location :** Michigan |
| (35) | 0:628  Males and immatures: 0:7 | **Years :** 1969-1978  **Location :** Connecticut |
| (42) | 0:88 | **Years :** 1981-1982  **Location :** Massachusetts |

| **Reference** | **MFIR *Ae. vexans*** | **YEAR/LOCATION** |
| --- | --- | --- |
| (38) | 1:48,481 | **Years :** 2003-2006  **Location :** North Dakota |
| (27) | 0:6,162 | **Years :** 2010-2011  **Location :** Connecticut |
| (13) | 1:17,521 | **Years :** 1997-2006  **Location :** Connecticut |
| (18) | 0:515  Males: 0:556 | **Years :** 1988-1989  **Location :** New York |
| (15) | 0:83,074 | **Years :** 1982-1984  **Location :** Indiana |
| (34) | 0:5,342 | **Years :** 1965  **Location :** Wisconsin |
| (32) | 0: 2,963 | **Years :** 1984-1989  **Location :** Michigan |
| (35) | 1:25,353  Males 0:1,794 | **Years :** 1969-1978  **Location :** Connecticut |
| (42) | 0:461 | **Years :** 1981-1982  **Location :** Massachusetts |

| **Reference** | **MFIR *An. punctipennis*** | **YEAR/LOCATION** |
| --- | --- | --- |
| (27) | 0:461 | **Years :** 2010-2011  **Location :** Connecticut |
| (13) | 1:1,457 | **Years :** 1997-2006  **Location :** Connecticut |
| (18) | 0:6 | **Years :** 1988-1989  **Location :** New York |
| (15) | 0:133 | **Years :** 1982-1984  **Location :** Indiana |
| (32) | 0:394 | **Years :** 1984-1989  **Location :** Michigan |
| (35) | *An*. spp. : 0:4,061  *An*. spp males and immatures: 0:67 | **Years :** 1969-1978  **Location :** Connecticut |
| (42) | 0:12 | **Years :** 1981-1982  **Location :** Massachusetts |

| **Reference** | **MFIR *An. earlei*** | **YEAR/LOCATION** |
| --- | --- | --- |
| (38) | 0:178 | **Years :** 2003-2006  **Location :** North Dakota |
| (35) | *An*. spp. : 0:4,061  *An*. spp. males and immatures: 0:67 | **Years :** 1969-1978  **Location :** Connecticut |

| **Reference** | **MFIR *An. quadrimaculatus*** | **YEAR/LOCATION** |
| --- | --- | --- |
| (27) | 0:105 | **Years :** 2010-2011  **Location :** Connecticut |
| (15) | 0:164 | **Years :** 1982-1984  **Location :** Indiana |
| (32) | 0:30 | **Years :** 1984-1989  **Location :** Michigan |
| (35) | *An. spp.* : 0:4,061  *An*. spp. males and immatures: 0:67 | **Years :** 1969-1978  **Location :** Connecticut |
| (42) | 0:2 | **Years :** 1981-1982  **Location :** Massachusetts |

| **Reference** | **MFIR *An. walkeri*** | **YEAR/LOCATION** |
| --- | --- | --- |
| (27) | 0:229 | **Years :** 2010-2011  **Location :** Connecticut |
| (13) | 1: 7,716 | **Years :** 1997-2006  **Location :** Connecticut |
| (32) | 0:340 | **Years :** 1984-1989  **Location :** Michigan |
| (35) | *An*. spp. : 0:4,061  *An*. spp. Males and immatures: 0:67 | **Years :** 1969-1978  **Location :** Connecticut |
| (42) | 0:1 | **Years :** 1981-1982  **Location :** Massachusetts |

| **Reference** | **MFIR *Cs. inornata*** | **YEAR/LOCATION** |
| --- | --- | --- |
| (38) | 1:7,348 | **Years :** 2003-2006  **Location :** North Dakota |
| (15) | 0:8 | **Years :** 1982-1984  **Location :** Indiana |
| (32) | 0:29 | **Years :** 1984-1989  **Location :** Michigan |
| (35) | *Cs*. spp: 0:21,646  *Cs*. spp males and immatures: 0:2,925 | **Years :** 1969-1978  **Location :** Connecticut |

| **Reference** | **MFIR *Cs. minnesotae*** | **YEAR/LOCATION** |
| --- | --- | --- |
| (27) | 0:2 | **Years :** 2010-2011  **Location :** Connecticut |
| (32) | 0:298 | **Years :** 1984-1989  **Location :** Michigan |
| (35) | *Cs*. spp: 0:21,646  *Cs*. spp. males and immatures: 0:2,925 | **Years :** 1969-1978  **Location :** Connecticut |

| **Reference** | **MFIR *Cs. morsitans*** | **YEAR/LOCATION** |
| --- | --- | --- |
| (13) | 1:2,495 | **Years :** 1997-2006  **Location :** Connecticut |
| (15) | 0:13 | **Years :** 1982-1984  **Location :** Indiana |
| (32) | 0:201 | **Years :** 1984-1989  **Location :** Michigan |
| (35) | *Cs*. spp: 0: 21,646  *Cs*. spp. Males and immatures: 0:2,925 | **Years :** 1969-1978  **Location :** Connecticut |
| (42) | 0:3 | **Years :** 1981-1982  **Location :** Massachusetts |

| **Reference** | **MFIR *Cq. perturbans*** | **YEAR/LOCATION** |
| --- | --- | --- |
| (38) | 0: 5,040 | **Years :** 2003-2006  **Location :** North Dakota |
| (27) | 1:4,791 | **Years :** 2010-2011  **Location :** Connecticut |
| (13) | 1:23,429 | **Years :** 1997-2006  **Location :** Connecticut |
| (18) | 0:2 | **Years :** 1988-1989  **Location :** New York |
| (15) | 0:2,458 | **Years :** 1982-1984  **Location :** Indiana |
| (34) | 0:500 | **Years :** 1965  **Location :** Wisconsin |
| (32) | 0: 29,574 | **Years :** 1984-1989  **Location :** Michigan |
| (35) | 1:26,666  Males and immatures: 0:143 | **Years :** 1969-1978  **Location :** Connecticut |
| (42) | 0:17 | **Years :** 1981-1982  **Location :** Massachusetts |

| **Reference** | **MFIR *Cx. pipiens/restuans*** | **YEAR/LOCATION** |
| --- | --- | --- |
| (27) | 1:2,207 | **Years :** 2010-2011  **Location :** Connecticut |
| (13) | 1:43,875 | **Years :** 1997-2006  **Location :** Connecticut |
| (15) | 0:985 | **Years :** 1982-1984  **Location :** Indiana |
| (34) | 0:227 | **Years :** 1965  **Location :** Wisconsin |
| (32) | 0:370 | **Years :** 1984-1989  **Location :** Michigan |
| (35) | *Cx*. spp: 0:24,702  *Cx*. spp. males and immatures: 0:4,481 | **Years :** 1969-1978  **Location :** Connecticut |
| (42) | 0:47 | **Years :** 1981-1982  **Location :** Massachusetts |

| **Reference** | **MFIR *Cx. territans*** | **YEAR/LOCATION** |
| --- | --- | --- |
| (27) | 0:23 | **Years :** 2010-2011  **Location :** Connecticut |
| (15) | 0:636 | **Years :** 1982-1984  **Location :** Indiana |
| (32) | 0:13 | **Years :** 1984-1989  **Location :** Michigan |
| (35) | *Cx*. spp: 0:24,702  *Cx*. spp. Males and immatures: 0:4,481 | **Years :** 1969-1978  **Location :** Connecticut |

| **Reference** | **MFIR *Ps. ferox*** | **YEAR/LOCATION** |
| --- | --- | --- |
| (27) | 0:1,771 | **Years :** 2010-2011  **Location :** Connecticut |
| (13) | 1:25,695 | **Years :** 1997-2006  **Location :** Connecticut |
| (15) | 0:26 | **Years :** 1982-1984  **Location :** Indiana |
| (32) | 0:1 | **Years :** 1984-1989  **Location :** Michigan |
| (35) | *Ps*. spp: 0:243  *Ps*. spp. males and immatures: 0:3 | **Years :** 1969-1978  **Location :** Connecticut |

| **Reference** | **MFIR *Ur. sapphirina*** | **YEAR/LOCATION** |
| --- | --- | --- |
| (27) | 0:605 | **Years :** 2010-2011  **Location :** Connecticut |
| (15) | 0:129 | **Years :** 1982-1984  **Location :** Indiana |
| (35) | 0:1,904  Males and immatures: 0:387 | **Years :** 1969-1978  **Location :** Connecticut |

| **Reference** | **MFIR *EXTRA SPECIES*** | **YEAR/LOCATION** |
| --- | --- | --- |
| (38) | *Cx. tarsalis*: 1: 3,636  *Ae. dorsalis*: 1: 7,015  *Ae. flavescens*: 1: 2,290  *Ae. melanimon*: 1: 45,325  *Ae. spencerii*: 0: 873  Other species not reported: 0: 8,084 | **Years :** 2003-2006  **Location :** North Dakota |
| (27) | *Ae. cantator:* 1:524  *Ae. aurifer:* 1:35  *Ae. sollicitans:* 0:11  *Ae. taeniorhynchus:* 0:4  *Ae. thibaulti:* 0:1,408  *An. crucians:* 0:5  *Cs. melanura:* 0:857  *Cx. salinarius:* 0:1,609  *Or. signifera:* 0:4  *Ps. ciliata:* 0:4  *Ps. columbiae:* 0:6 | **Years :** 2010-2011  **Location :** Connecticut |
| (13) | *Cs. melanura*: 1:65,703  *Ae. aurifer*: 1:1,509  *Ae. cantator*: 1:880  *Ae. sollicitans*: 1:3,027  *Ae. taeniorhynchus*: 1:5,315 | **Years :** 1997-2006  **Location :** Connecticut |
| (18) | *Ae. decticus*: 0:54 | **Years :** 1988-1989  **Location :** New York |
| (15) | *Cx. erraticus/peccatur*: 0:36  *Cx. salinarius*: 0:45  *Ps. ciliata*: 0:10  Some other non-mosquito flies tested as well, all negative | **Years :** 1982-1984  **Location :** Indiana |
| (34) | *Cx. salinarius:* 0:108  *Ae*. spp. : 0:4,280 | **Years :** 1965  **Location :** Wisconsin |
| (25) | *Ae. cantator*: 1:4,474 | Review paper, 1990 |
| (32) | *Ae. aurifer*: 1:421  *Ae. decticus*: 0:18  *Ae. dorsalis*: 0:3  *Cx. salinarius*: 0:3  *Cx. tarsalis*: 0:2  *Cs. impatiens*: 0:2 | **Years :** 1984-1989  **Location :** Michigan |
| (35) | *Ae. aurifer*: 0: 615  *Ae. cantator*: 1:4,024  *Ae. sollicitans*: 0: 1,475  Males and immatures:  *Ae. aurifer*: 0:2  *Ae. cantator*: 0:114  *Ae. sollicitans*: 0:5 | **Years :** 1969-1978  **Location :** Connecticut |
| (42) | N/A | **Years :** 1981-1982  **Location :** Massachusetts |
